# Evaluation of Wastewater Treatment by Microcosms of Vertical Subsurface Wetlands in Partially Saturated Conditions Planted with Ornamental Plants and Filled with Mineral and Plastic Substrates

**DOI:** 10.3390/ijerph16020167

**Published:** 2019-01-09

**Authors:** Luis Sandoval, José Luis Marín-Muñiz, Sergio Aurelio Zamora-Castro, Fabiola Sandoval-Salas, Alejandro Alvarado-Lassman

**Affiliations:** 1 División de Estudios de Posgrados e Investigación, Tecnológico Nacional de México/Instituto Tecnológico de Orizaba, Oriente 9 852, Emiliano Zapata Sur, C.P. 94320 Orizaba, Veracruz, Mexico; lcsandovalh@gmail.com; 2 Department of Civil Engineering, Tecnológico Nacional de México/Instituto Tecnológico Superior de Misantla, Km. 1.8 Carretera a Loma del Cojolite C.P. 93821 Misantla, Veracruz, Mexico; 3 El Colegio de Veracruz, Carrillo Puerto No. 26, 91000 Xalapa, Veracruz, Mexico; soydrew@hotmail.com; 4 Facultad de Ingeniería, Universidad Veracruzana Bv. Adolfo Ruíz Cortines 455, Costa Verde, C.P. 94294 Boca del Rio, Veracruz, Mexico; szamora@uv.mx; 5 División de Estudios de Posgrados e Investigación, Tecnológico Nacional de México/Instituto Tecnológico Superior de Perote, C.P. 91270 Perote, Veracruz, Mexico; investiga.itspe@gmail.com

**Keywords:** PET, ornamental plants, porous river stone, constructed wetlands, microcosms, wastewater

## Abstract

The current knowledge about the role terrestrial ornamental plants play in constructed wetlands (CWs) has scarcely been evaluated. Likewise, little attention has been given towards the use of new support or fill media for subsurface flow CWs, which may result in the reduction of costs when implemented on a large scale. This study evaluated, during nine months, the effect of three terrestrial ornamental plants and two substrates on the elimination of pollutants in wastewaters by using fill-and-drain vertical subsurface flow CWs (FD-CWs). Sixteen microcosms were used, nine filled with polyethylene terephthalate (PET) and nine with porous river stone (PRS). For each type of substrate, duplicates of microcosms were used, utilizing *Anthurium* sp., *Zantedeschia aethiopica*, and *Spathiphyllum wallisii* as vegetation and two other CWs without vegetation as controls. The environmental conditions, number of flowers, and height of the plants were registered. The results revealed that both substrates in the FD-CWs were efficient in removing pollutants. The average removal of pollutants in systems with vegetation revealed a positive effect on the reduction of the biochemical oxygen demand (55–70%), nitrates (28–44%), phosphates (25–45%), and fecal coliforms (52–65%). Meanwhile, in units without vegetation, the reduction of pollutants was nearly 40–50% less than in those with vegetation. The use of PET as a filling substrate in CWs did not affect the growth and/or the flowering of the species; therefore, its use combined with the species studied in CWs may be replicated in villages with similar wastewater problems. This may represent a reduction in implementation costs when utilizing PET recycled wastes and PRS as substrates in these systems in comparison with the typical substrates used in CWs. More studies are needed to better understand the interactions among these novel support media and the commercial terrestrial ornamental plants.

## 1. Introduction

Achieving sustainability of the decentralized use of wastewaters is a challenge developing countries are currently facing [1,2]. However, current treatment procedures are expensive, and they require high energy costs and highly-trained personnel for operation and maintenance [3]. Consequently, in low-income and lower-middle countries, wastewater runoff without treatment occurs for up to 72% and 92%, respectively, of the total waste generated [4], and this runoff is finally disposed mainly in surface bodies of water, threatening the health of these recipient systems [5].

Nowadays, constructed wetlands (CWs) are considered an ideal environmental technology for the treatment of domestic and industrial wastewater due to their low cost of implementation and operation, zero energy consumption, and great results in the elimination of pollutants [6,7]. Such systems work by imitating natural wetlands’ characteristics in the biochemical interactions among vegetation, substrates, and microorganisms [8]. In subsurface conditions, depending on the water direction, the CWs are called horizontal or vertical subsurface CWs. The nitrification processes are usually higher in the vertical subsurface flow wetlands than horizontal subsurface flow wetlands [9,10]; in the vertical subsurface flow wetlands, the denitrification and COD (Chemical Oxygen Demand) removal processes are usually more accelerated [11,12]. One possible option to solve the wastewater treatment in a single system is through the use of a new design of CWs called partially saturated vertical wetlands; these are similar to the tidal flow CWs, where the bed is intermittently saturated, which is related to the fill and drain phases (FD-CWs). Such systems are mostly applied when increased oxygen transfer is needed [13] but may be used to promote anaerobic processes when the holding phase is extended. The shortcomings of the FD-CWs are that the conditions in the system are not constantly anaerobic as compared to, for example, the upflow CWs. It is thus necessary to evaluate the viability of the FD-CWs using ornamental plants for the treatment of wastewater. The aforementioned benefits have not been enough for these systems to be fully utilized in intertropical countries like Mexico [14].

On the other hand, the substrates or the filling material that work as filters in subsurface flow CWs constitute 50% of the system volume [15]. In addition, they provide a support media to the vegetation sown in them, and they also allow biological and physicochemical interactions. Moreover, they are the main media in which biofilms that noticeably contribute to removing pollutants are developed [16]. The most used mineral substrates in CWs are traditional substrates, such as sand and gravel [17,18]. They usually imply a cost increase when they are implemented in actual-size CWs due to the second industrial uses that these materials have in industries such as construction; therefore, cost is one of the main limiting factors that many people in developing countries face when they try to adopt these sustainable technologies, especially in rural remote zones with wastewater treatment problems and low income [19]. Studies focused on the selection of new substrates from local material or from the reuse of other material are necessary to promote the utilization of cheaper but equally functional substrates in CWs, such as polyethylene terephthalate (PET) wastes [8,17].

Vegetation plays a crucial role in the pollutant removal process; hence, the plants selected must be able to adapt to the conditions of the CWs and must be suitable for the removal of pollutants. However, it is a subject that has been little studied in tropical and intertropical areas [20]. Generally, the plants used in CWs are macrophytes typical of natural wetlands, such as *Phragmites australis* and species of the *Typha*, *Scirpus*, and *Cyperus* genera [6]. Few studies have considered the selection of different species of plants that also promote other benefits, such as the ornamental and commercial uses of the flowers that may be produced in the CW plants [21].

Most of the studies performed regarding the selection or examination of new plants have been principally developed in warm climates and, to a lesser extent, in tropical climates [22], where the temperature is warmer and the sunlight is stronger. This may favor the ideal conditions for fast growth, and the plants may mainly contribute to a meaningful absorption of nutrients from the wastewaters without treatment [23,24]. The vegetation favors oxygenation of the substrate and, thus, development of the aerobic bacteria population. In tropical and subtropical countries, such as China, Thailand, Portugal, Italy, and Mexico, the initiative of producing flowers in CWs has been developed with esthetic and economic purposes in mind [25,26,27,28].

Due to these aforementioned facts, the research related to CWs employed for wastewater treatment has notably increased in the literature [29,30]. Nevertheless, the use of new substrates with lower commercial value [8], ornamental plants that favor the insertion of these ecotechnologies in rural landscapes in an aesthetic form, and the biochemical interactions that occur in the CW systems have scarcely been studied [31].

In this sense, inquiries focused on the search for new lower-cost substrates are important, as well as materials for use as substrates in CWs that are easy to obtain and that do not compromise the processes of pollutant removal. One of these materials may be rough residues of polyethylene terephthalate (PET) bottles; these rough surfaces can favor the habitat for development of bacterial biofilms that are the main participants in the removal of pollutants in CWs. The implementation of PET coarse wastes as substrates in CWs is expected to give a new functional use to this material. In 2014, in the USA there were 8200 million kilograms of PET bottles and containers potentially available to be recycled, of which 2700 million kilograms were recycled, thus resulting in a recycling gross rate of 31.0% [32]. Additionally, in Mexico, an average of 90 million bottles used for bottling water and soft drinks are thrown away on beaches, in forests, and in the streets [33]. The aforementioned situation may endanger the environment, considering that the biodegradation period for these bottles is 500 years on average [34]. If the coarse sections of PET are considered or a pretreatment is implemented on the smooth surfaces, they may favor the development of a bacteria population that is fundamental to the bioremediation processes when used as filling material in CWs [18].

The aim of this study was to evaluate the use of FD-CWs in the removal of pollutants and the importance of ornamental plants (*Anthurium* sp., *Zantedeschia aethiopica*, and *Spathiphyllum wallisii*) and the use of mineral (porous river stone) and plastic (PET) wastes as substrate materials in wastewater treatment using CWs. 

## 2. Materials and Methods

### 2.1. Field of Study

This study was carried out between June 2016 and March 2017 in the municipality of Misantla, located in the mountainous central-northern area of the State of Veracruz, Mexico (19°56’ N and 96°51’ W). The climate in this area is classified as being semi-warm humid with rains occurring all year (45%), warm humid with rains occurring all year (38%), and warm humid with abundant rains in summer (17%), with the highest temperatures registered in June and the lowest in January. The altitude range of the basin is 10–1900 m above sea level [35]. The average annual rainfall is between 1900 and 2100 mm, and the average annual temperature is 20–26 °C. The main economic activities are agriculture and stockbreeding [36].

### 2.2. Characteristics of the FD-CWs

**Figure 1:**
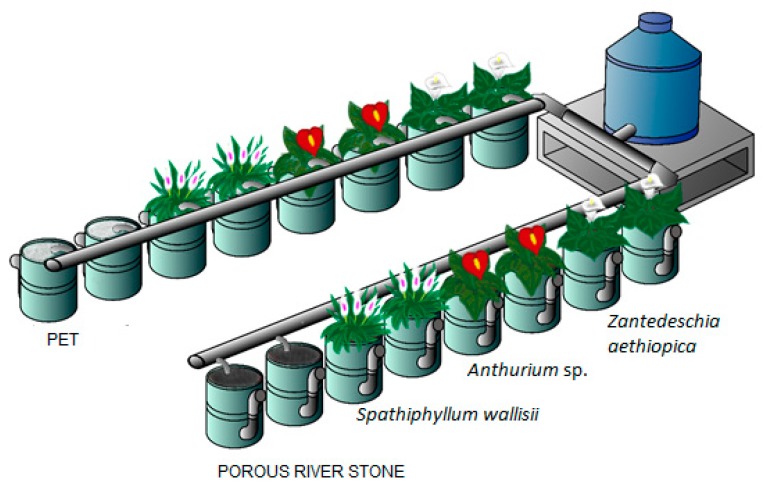
Configuration of wasteland microcosm.

The systems utilized as FD-CW microcosms during the experimentation were 16 plastic cylinders fed with a vertical flow (29 cm diameter, 36 cm height, 20 L volume) (Figure 1). Porous river stone (PRS) was used as substrate. It was taken from the river Misantla, with a diameter of 2.5 cm and a slightly homogeneous surface with a mean porosity of 0.5. For PET substrate, thick sections of recycled bottles were used; these bottles had been utilized for bottling water and soft drinks, with diameters from 3 to 5 cm. In this study, PET fulfilled the function of a support means for vegetation and habitat for development of bacterial biofilms, with a mean porosity of 0.8. The experimental units were filled from the bottom to a level of 26 cm with the respective substrates employed. In the experimental units filled with PET, 10 cm of PRS were added to prevent the PET from floating over the water outlet; this layer did not work as treatment media. The water supply in the microcosms of wetlands was made with the hydraulic flow equation (HF):

HF = (V × d)/ hydraulic retention time (HRT)

V = Volume of the cell (L), (17.174 L)

d = Porosity or space available for water flow through the wetland (percentage expressed as decimal) (0.5 PRS and 0.8 PET)

where HRT is the hydraulic retention time of three days, and three different species of ornamental plants were used: *Anthurium* sp., *Spathiphyllum wallisii*, and *Zantedeschia aethiopica* (14–19 cm height). One individual per experimental unit was planted according to the arrangement presented in Figure 1. For the selection of the vegetation employed, the plants had to be able to adapt, and their vascular height had to allow them to survive in flooding conditions and be resistant to weathering (wind, rain). Additionally, the plants had to be of commercial interest. In this way, the systems may be employed as culture media [8,37]. The ornamental plants used were acquired in two ways: *Spathiphyllum wallisii* and *Zantedeschia aethiopica* were collected from areas near the study locations to favor their suitability to local conditions, and *Anthurium* sp. was acquired in a local nursery in its flowering period at the time of planting. Duplicates of every experimental unit in both substrates (PET and PRS) and plants (Figure 1) as well as two microcosms filled with every substrate were used as controls without vegetation. Fill-and-drain mode in the CWs consisted of two phases: filling phase and draining phase (four hours every three days).

In the first 30 days after planting, the CWs were fed with potable water. From day 31, a proportion of domestic wastewaters (originating from a small neighborhood in Misantla, Veracruz, México) was added. For adapting the vegetation to the new conditions of water quality, a 1500-L tank was stocked up every two days with an electric peripheral water pump (TruperMR of ½ HP, Siemens 3301, New York, NY, USA). Before entering the tank, the collected wastewater that fed the microcosms crossed a sieve mesh with an opening of 0.074 mm to reduce the solids content. 

### 2.3. Experimental Design

The CWs were evaluated with an experimental design of two factors, factor one was a plant species (*Anthurium* sp., *Zantedeschia aethiopica*, and *Spathiphyllum wallisii*) seeded in duplicates and factor two was the substrate type (Sixteen microcosms, nine were used filled with polyethylene terephthalate (PET) and nine with porous river stone (PRS)) of which two of each substrate served as controls without vegetation.

### 2.4. Sampling and Analysis

From day 31, the tank was fed with 100% wastewater and during the period from 30 June 2016 to 12 March 2017, every 15 days one sample was taken from the influent and the effluent of each of the CWs. The samples were analyzed at a laboratory the Higher Technological Institute of Misantla (ITSM). The biochemical oxygen demand (BOD_5_), nitrates (N-NO_3_), phosphates (P-PO_4_), and fecal coliforms (FCs) were determined in duplicates by standard methods [38]. Total dissolved solids, electrical conductivity (EC), pH, and water temperature were measured with a Hanna H198194 multiparameter meter (Hanna Instruments, Woonsocket, RI, USA) at the influent and the effluent of the microcosms. In addition to these data, every 15 days the environmental temperature was recorded every 30 min between 9:00 and 10:00 h and between 14:00 and 15:00 h. The mean of each measurement was estimated and recorded. The height of the plant, measured with a measuring tape, and the number of the flowers were registered every 30 days.

### 2.5. Statistical Analysis

All statistical analyses were performed using Minitab version 16.1.0 (Minitab Inc., State College, PA, USA). The standard error for pH, EC, total dissolved solids (TDS), BOD_5_, nitrates, phosphates, and FC, as well as the height of the plant and the number of flowers, was found for the treatment of each microcosm. The differences between the results of the treatments were estimated by two- way repeated measures analysis of variance for the 18 measurement periods. For all tests, a significance level of *p* < 0.05 was applied. Due to the data failing a normality test and equal variance test, a repeated measures ANOVA on ranks was performed.

## 3. Results and Discussion

**Figure 2:**
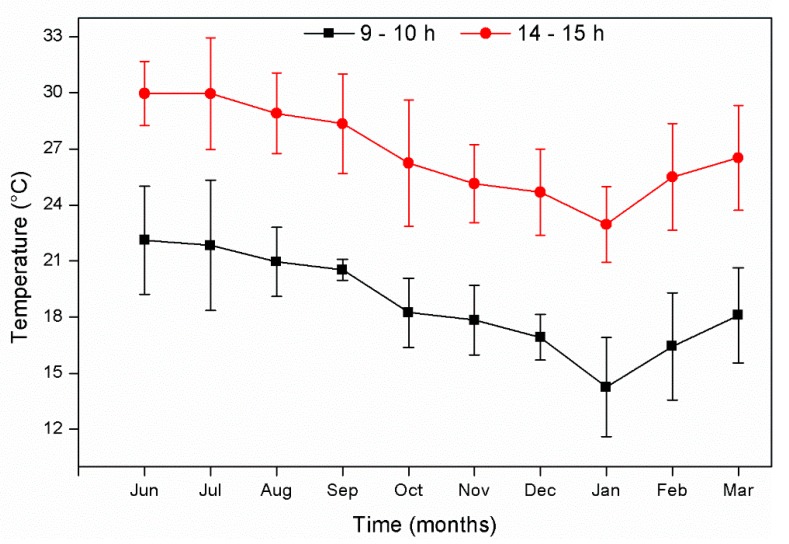
Environmental temperatures registered at 9–10 h and 14–15 h during the experimental period. Values are given as the mean ± standard error.

The environment’s temperature oscillated between 14 and 30 °C (Figure 2). This temperature is ideal for pollutant removal in CWs, as bacterial activity increases at a temperature > 20 °C [15,39], as shown in this study (Table 1). 

The influent’s mean pH value was 7.2, and the mean pH value in the units with no vegetation was 8.0. In the CWs with vegetation, the pH value diminished (6.8–7.1). That value is in line with the findings of Vymazal [40], who indicated that values tend to decrease in vertical-flow wetlands due to greater oxygenation of the substrates in comparison with horizontal-flow systems, and Jin [40], who indicated that bacteria are sustained by the nitrification processes, an initial step towards nitrogen removal when ammonium predominates and causes pH reduction. The mean EC was found to be 1306.45 ± 52.07 µS·cm^−1^, with a diminished value in CWs with vegetation; in contrast, CWs with no vegetation showed values between 1006.49 and 1196.96 µS·cm^−1^. According to statistical analyses, the EC significantly decreased (*p* ≤ 0.05) in the assessed systems, with similar values in the effluents (Table 1). This decrease in regard to the influent is explained by the removal of roots due to absorption, detritus, and sedimentation of suspended particles [41,42]. With regard to suspended solids, a decrease was found relative to the value of the influent (267.59 ± 5.94 mg·L^−1^), and in the effluent the value oscillated between 168.47 and 193.89 mg·L^−1^; statistically, it significantly decreased in relation to the system’s influent (*p* > 0.05). The reported values in this study are among the allowable limits set by the EU (USEPA), which marks it as 500 mg·L^−1^, and our results are consistent with reports from other studies, in similar conditions, that employed vertical-flow wetlands in which reduced values were found after the treatment [14].

### 3.1. Plant Growth and Flower Production in Microcosms

**Figure 3:**
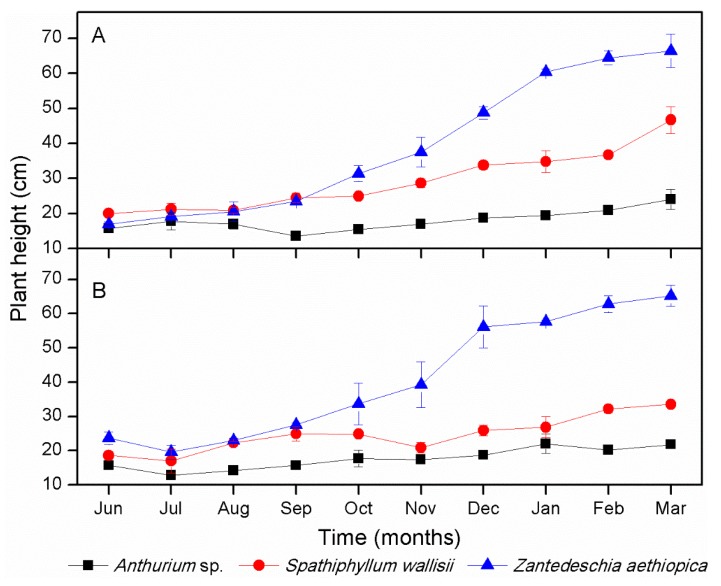
Plant growth of *Anthurium* sp., *Spathiphyllum wallisii*, and *Zantedeschia aethiopica* registered in polyethylene terephthalate (PET) (**A**) and porous river stone (PRS) (**B**) substrates. Values are given as the mean ± standard error.

Plant growth during nine months of work is shown in Figure 3. For the *Zantedeschia aethiopica,* the greatest growth was observed in both substrates (PET and PRS), with a maximum height of 65.1 cm and 60.3 cm with PRS and PET as support media, respectively. For *Spathiphyllum wallisii*, growth of 40.1 cm and 27.8 cm was observed in PRS and PET, respectively, showing significant differences (*p* = 0.036) between support media at the end of the study (March 2017). The lowest growth percentage was shown by *Anthurium* sp., which reached a maximum height of 18.5 cm and 17.8 cm in PRS and PET, respectively. No significant differences were found with regard to growth in different support media (*p* = 0.021). Some of the leaves of this plant dried and regrew; in this sense, the shown growth is not observable due to this phenomenon. Even though the PET substrate only affected the growth of *S. wallisii* in comparison with the use of PRS, such species along with *Z. aethiopica* had greater growth development than *Anthurium* sp. The growth of the latter was slow, but was important to consider the assessment of this species in the system due to its aesthetic characteristics and higher economic value ($7–14 per plant with flowers) in comparison to the two other species ($2–9 per plant with flowers). 

Regarding flower production during the study period (Table 2), significant differences (*p* < 0.05) were found among plants. The species that produced the most flowers was *Spathiphyllum wallisii*, with an average of 12 flowers in PET and nine in PRS, followed by *Zantedeschia aethiopica*, producing 10 flowers in PET and seven in PRS on average; *Anthurium* sp. was able to produce only two flowers throughout the whole study in both PET and PRS. Although *S. wallisii* had a lower growth in PET (Figure 3B), it had greater flower production, which is an important benefit of this species. Thus, even though PET does not favor the best growth of the species, it favors greater flowering. For the two other species, both the growth and the flowering had similar behavior.

**Figure 4:**
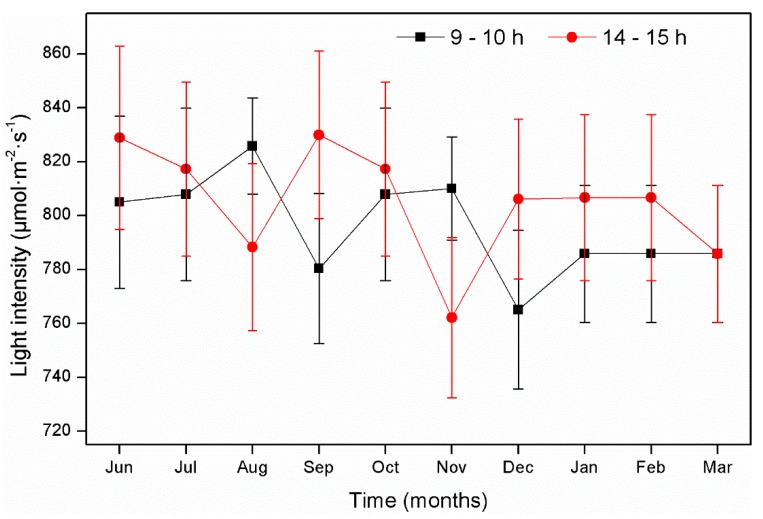
Light intensity registered at 9 to 10 h and 14 to 15 h during the experimental period. Vertical bars represent the standard error of the mean; values are given as the mean ± standard error.

**Figure 5:**
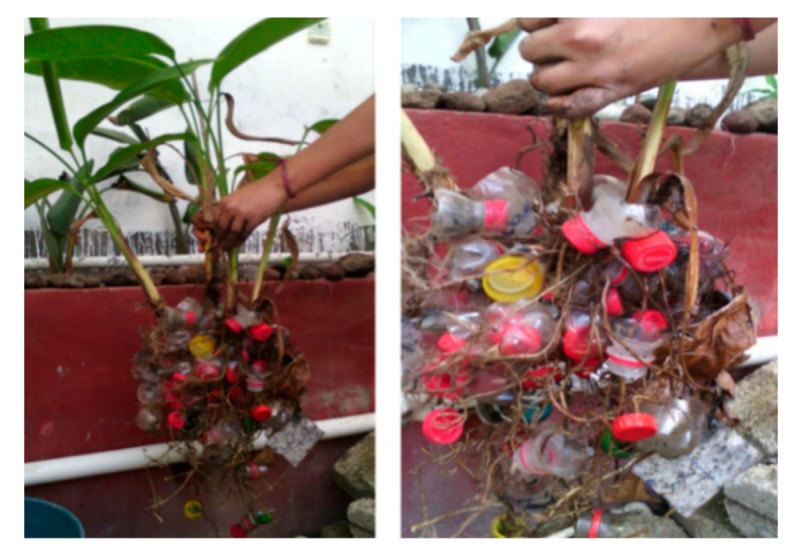
Radical development in PET substrate.

Plant growth was favored by the light intensity conditions during the study (Figure 4), with values between 720 to 856 µmol·m^−2^·s^−1^. These values concur with those reported by Olguín et al. [43], who found similar ranges for plant growth in tropical climates. Figure 5 provides an example showing how the plants developed in the CWs utilizing PET as a substrate. Our results indicated adequate growth and demonstrate that healthy plants used approximately 20% of pollutants from the wastewater in order to grow. This information concurs with that reported by Vymazal [44], who indicated that plants absorb 14–20% of wastewater pollutants, on average, for their development. This suggests that PET is a support medium that can be used in future CW designs due to its ability to obtain desirable results in vegetal development and ornamental species blooming.

### 3.2. Pollutant Concentration and Removal in Microcosms

**Figure 6:**
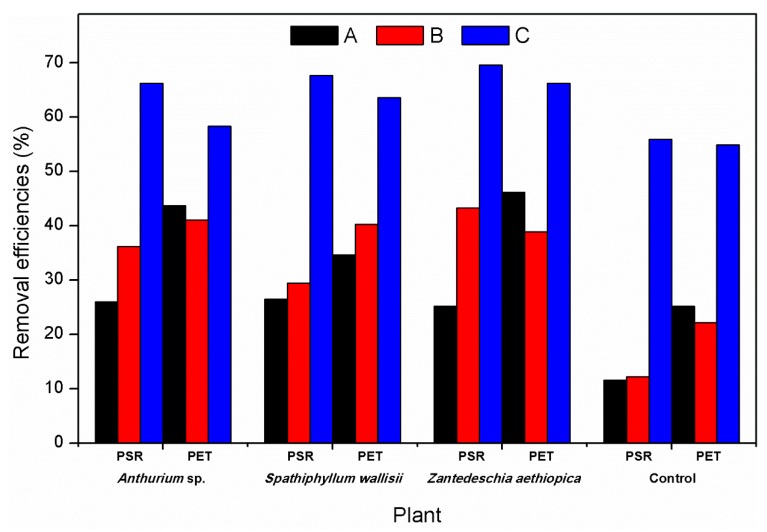
Pollutant removal of P-PO_4_ (phosphates) (**A**), N-NO_3_ (nitrates) (**B**), and BOD_5_ (five-day biochemical oxygen demand) (**C**) in influent and effluent of microcosms.

Vegetation in CWs plays an important role in the removal of pollutants. For N-NO_3_ in the studied CWs, the influent was found in ranges of 12.08 ± 0.36 mg·L^−1^ (Table 3). This value is above the allowable limits (10 mg·L^−1^) according to Meng et al. [45], and because of the toxicity of the nitrates and nitrites, water consumption is not safe with values above the permissible value. These levels decreased in systems with vegetation in both PET (7.24 ± 0.19 mg·L^−1^) and PRS (7.69 ± 0.14 mg·L^−1^), whereas in units without vegetation, the values were 9.40 ± 0.35 mg·L^−1^ and 10.59 ± 0.12 mg·L^−1^ for the PET and PRS substrates, respectively, indicating a phytoremediation effect. In Figure 6, the N-NO_3_ removal efficiency is shown throughout the study period. A removal of 29% and 43% (*p* ≤ 0.001) was observed for the systems with vegetation, in comparison with controls without vegetation, which ranged between 12% and 22%. Removals did not have significant variations between the two kinds of substrates utilized in this study. Such results highlight the utility of both substrates for CW design. 

The removal differences observed within microcosms with plants and microcosms without vegetation indicate that in microcosms with plants there were microsites that favored nitrification and other anoxic microsites in which nitrate was denitrified. A greater removal in the presence of plants is attributed to the natural function of plants of providing carbon exudates from the root zone that intensify denitrification [46]. With regard to P-PO_4_, the influent’s concentration was 11.89 ± 0.38 mg·L^−1^ (Table 3). Average P-PO_4_ values of 6.95 ± 0.36 mg·L^−1^ and 8.73 ± 0.27 mg·L^−1^ were found in PET and PRS, respectively, whereas average P-PO_4_ values of 8.89 ± 0.27 and 10.51 ± 0.26 mg·L^−1^ were observed for systems with vegetation and for systems without vegetation, respectively. For P-PO_4_ removal (Figure 6), it was found that planted wetlands in a PRS media were more efficient in regard to P-PO_4_ removal (*p* ≤ 0.0001) compared to PRS media without plants (10.51 ± 0.26 mg·L^−1^). Although the retention of phosphorus forms is considered one of the most important attributes of CWs, the differences observed according to the absence/presence of plants is related to the plant uptake [47] reflected in the growth of the vegetation. The removal values were between 35% and 46% for wetlands with vegetation, and for wetlands without vegetation with PRS substrate the removal values were 25%. The obtained results are similar to those reported by Prochaska and Zouboulis [48], who had employed a mixture of river sand and limestone as substrate, with removal values up to 45% observed for CWs with vegetation. For wetlands with vegetation in the PET substrate, no significant differences were found among plants (*Anthurium* sp., *Zantedeschia aethiopica*, and *Spathiphyllum wallisii*), with averages of 25.5%; in controls with vegetation, the average was 12%. This result seems to be optimal in terms of water quality for farming irrigation, in which the phosphates could be employed to sustain crops [49]. This shows that certain plants favor pollutant removal processes just as certain support media do [50,51]. Research studies must focus on PET assessment, along with other support media, such as PRS, gravel, and tezontle, that favor removal processes, as well as the reduction of the substrates’ size, in order to provide a better contact surface between water and the vegetation’s support media [27].

The initial BOD_5_ concentration (Table 3) was 115.96 ± 1.85 mg·L^−1^ in the influent, whereas in the effluent, regardless of the support media, the concentrations diminished to values ranging from 35.21 ± 1.56 to 48.31 ± 2.149 mg·L^−1^. In units without vegetation, the concentrations were 52.30 ± 2.88 mg·L^−1^ in PET and 51.12 ± 2.20 mg·L^−1^ in PRS. For the DBO_5_, no significant differences were found (*p* = 0.734) with respect to the substrates. Figure 6 shows the results for this variable of 58–70% in systems with vegetation regardless of the support media, whereas in controls without vegetation, the average removal was 55.5% for both media. Similar results were observed in other studies showings that systems with vegetation favor the BOD_5_ removal process [52,53].

**Figure 7:**
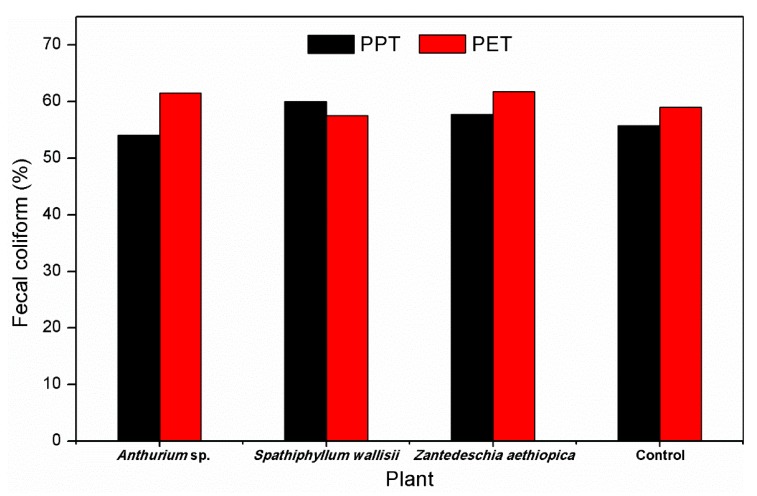
Fecal coliform removal in influent and effluent of microcosms.

Regarding the total FCs, results are presented in Table 3 with respect to the FC concentration, which significantly diminished (*p* = 0.05) in all systems with or without vegetation, regardless of the substrate. FC removal results (Figure 7) oscillated between 54% and 62%. These results are lower than those reported by García et al. [14], who obtained removal results in systems with and without vegetation between 65% and 92%, whereas Vymazal [39] obtained removal averages of 91% and 97%. This could be due to the reduction of temperature in wetlands, which increases the lifespan of coliforms in wetlands and the HRT used in this study, which was three days, while reports from different authors estimate it as being between four and five days [39,27,54]. Furthermore, the presence of high concentrations of nutrients favors the development of FCs [55]. Thus, it is necessary to consider increasing the HRT if the purpose is the removal of FCs from wastewater. 

The results obtained in this study are consistent with those reported by different authors who have employed systems with and without vegetation in order to assess their efficiency. The results indicate that the presence of vegetation favors pollutant removal processes due to the development of aerobe microorganisms, which noticeably contribute to the removal of pollutants [25,56]. 

## 4. Conclusions

The results of the present study show that in wetland microcosms with partially saturated conditions, PET wastes are a potentially valid form of substrate media that function similarly to PRS, a more common material used in CWs. The plants were easily adapted in PET substrate and the removal percentages of pollutants were similar to those detected in PRS medium. Both support media notably contributed to the reduction of typical wastewater pollutants such as N-NO_3_, P-PO_4_, and BQD_5_ and microorganisms such as FCs.

The use of *Anthurium* sp., *Zantedeschia aethiopica*, and *Spathiphyllum wallisii* in the treatment systems was shown to increase the systems’ removal efficiency by up to 30% in the main parameters evaluated, compared with units without plants, while providing an aesthetic value to the systems, thus allowing their insertion into landscapes in an aesthetically pleasing way. Furthermore, the production of commercial flowers would permit maintenance work to be compensated with an economic incentive to the caretakers and handlers of the systems that treat wastewater. Additionally, the water treated could be used for green spaces and agriculture, since this effluent fulfils the quality parameters considered acceptable for these purposes.

The functionality of PET as a substrate material may indicate the potential for a significant cost reduction if it is implemented instead of using other materials that require higher investment costs (up to 80% less). It may also represent a considerable contribution to reducing the impact caused by PET in the environment. Therefore, when considering the potential benefits of both the use of PET substrates and the production of flowers in constructed wetlands, the use of PET and ornamental species should be considered in future designs in landscape engineering treating municipal wastewater. Although this study emphasizes the potential of three ornamental plants in monocultures in removing pollutants from domestic wastewater, it is suggested that future research projects should study the same species in polycultures, with the aim to detect better removals of pollutants and promote better aesthetics of the systems and thus to promote the use of flower boxes (planters) as treatment systems.

## Figures and Tables

**Table 1:** Wetland plants in different substrates. Chemical parameters at the inlet and outlet of wetland microcosms.

Parameters	Influent	*Anthurium* sp. PET	*Spathiphyllum wallisii* PET	*Zantedeschia aethiopica* PET	*Anthurium* sp. PRS	*Spathiphyllum wallisii* PRS	*Zantedeschia aethiopica* PRS	Control PET	Control PRS
Water temperature (°C)	23.34 ± 0.70	17.14 ± 0.17	16.99 ± 0.21	17.44 ± 0.19	17.18 ± 0.20	16.99 ± 0.16	14.44 ± 0.32	17.61 ± 0.16	17.48 ± 0.08
pH	7.9 ± 0.12	7.86 ± 0.06	7.70 ± 0.06	7.09 ± 0.05	7.40 ± 0.06	7.12 ± 0.06	7.17 ± 0.06	7.19 ± 0.06	7.04 ± 0.05
EC (µS·cm^−1^)	1306.45 ± 52.07	1116.98 ± 36.01	1167.94 ± 28.75	1118.48 ± 29.22	1019.78 ± 26.88	1080.12 ± 26.08	1032.80 ± 27.60	1050.2 ± 43.51	1042.26 ± 31.18
TDS (mg·L^−1^)	267.59 ± 5.94	169.55 ± 2.00	183.71 ± 1.67	168.50 ± 2.18	178.82 ± 1.28	183.82 ± 1.54	168.509 ± 2.35	190.43 ± 3.46	212.99 ± 2.92

Values are given as the mean ± standard error (*n* = 36). PET: polyethylene terephthalate; PRS: porous river stone; EC: electrical conductivity; TDS: total dissolved solids.

**Table 2:** Flower production in microcosms.

	Total Flowers		
	*Anthurium* sp.	*Spathiphyllum wallisii*	*Zantedeschia aethiopica*
PET	2	12	10
PRS	2	9	7

**Table 3:** Water quality parameters in both the influent and effluent as well as parameter removal in microcosms.

Vegetation Used in Microcosms								
Parameters	*Anthurium* sp. PET	*Spathiphyllum wallisii* PET	*Zantedeschia aethiopica* PET	*Anthurium* sp. PRS	*Spathiphyllum wallisii* PRS	*Zantedeschia aethiopica* PRS	Control PET	Control PRS
BOD_5_								
Influent concentration (mg·L^−1^)	115.96 ± 1.85	115.96 ± 1.85	115.96 ± 1.85	115.96 ± 1.85	115.96 ± 1.85	115.96 ± 1.85	115.96 ± 1.85	115.96 ± 1.85
Effluent concentration (mgL^−1^)	48.31 ± 2.19	42.20 ± 2.26	36.41 ± 2.00	39.15 ± 1.81	37.40 ± 2.03	35.21 ± 1.56	52.30 ± 2.88	51.12 ± 2.20
N-NO_3_								
Influent concentration (mg·L^−1^)	12.08 ± 0.36	12.08 ± 0.36	12.08 ± 0.36	12.08 ± 0.36	12.08 ± 0.36	12.08 ± 0.36	12.08 ± 0.36	12.08 ± 0.36
Effluent concentration (mg·L^−1^)	7.12 ± 0.19	7.22 ± 0.18	7.38 ± 0.21	7.71 ± 0.14	8.52 ± 0.11	6.85 ± 0.18	9.40 ± 0.35	10.59 ± 0.12
P-PO_4_								
Influent concentration (mg·L^−1^)	11.89 ± 0.38	11.89 ± 0.38	11.89 ± 0.38	11.89 ± 0.38	11.89 ± 0.38	11.89 ± 0.38	11.89 ± 0.38	11.89 ± 0.38
Effluent concentration (mg·L^−1^)	6.69 ± 0.44	7.77 ± 0.31	6.40 ± 0.34	8.80 ± 0.26	8.74 ± 0.27	8.66 ± 0.27	8.89 ± 0.27	10.51 ± 0.26
FC								
Influent concentration (MPN·100 mL^−1^)	3319.31 ± 64.41	3319.31 ± 64.41	3319.31 ± 64.41	3319.31 ± 64.41	3319.31 ± 64.41	3319.31 ± 64.41	3319.31 ± 64.41	3319.31 ± 64.41
Effluent concentration (MPN·100 mL^−1^)	1277.23 ± 94.71	1409.95 ± 84.72	1267.07 ± 95.54	1523.81 ± 90.05	1326.72 ± 102.79	1403.13 ± 93.22	1360.43 ± 89.01	1469.30 ± 90.22

Values are given as the mean ± standard error (*n* = 36); different letters indicate significant differences between the columns at the 50% significance level. PRS: porous river stone; PET: polyethylene terephthalate; P-PO_4_: phosphates; N-NO_3_: nitrates; BOD_5_: five-day biochemical oxygen demand; FC: fecal coliform. Throughout the study after 30 days of adaptation of the systems.
